# Magic of a Common Sugar Pill in Cancer: Can Metformin Raise Survival in Pancreatic Cancer Patients?

**DOI:** 10.7759/cureus.16916

**Published:** 2021-08-05

**Authors:** Mallika Gyawali, Nanditha Venkatesan, Opemipo D Ogeyingbo, Renu Bhandari, Rinky A Botleroo, Roaa Kareem, Rowan Ahmed, Abeer O Elshaikh

**Affiliations:** 1 Internal Medicine, California Institute of Behavioral Neurosciences & Psychology, Fairfield, USA; 2 Internal Medicine, All India Institute of Medical Sciences, Raipur, IND; 3 Internal Medicine, Saint James School of Medicine, Park Ridge, USA; 4 Public Health, Walden University, Minneapolis, USA; 5 Internal Medicine, Manipal College of Medical Sciences, Pokhara, NPL

**Keywords:** pancreatic cancer, diabetes mellitus, metformin, antidiabetic drugs, survival

## Abstract

Pancreatic cancer is one of the common cancers globally, with a poor survival outcome. Metformin, a popular anti-diabetic drug, has gained popularity for its use in the chemoprevention of cancer. However, results regarding the survival benefit of metformin in pancreatic cancer have been unpredictable. In this review, we aim to analyze the use of metformin in pancreatic cancer patients with pre-existing diabetes mellitus for survival benefit. We systematically conducted a literature search in PubMed, Science Direct, and Scopus databases to collect the relevant articles and reviewed them. Eventually, 11 quality appraised articles were included accessing overall survival as the primary outcome. Our results concluded that metformin can efficaciously improve survival in pancreatic cancer patients with coexisting diabetes mellitus, but the results are still incongruent. Hence, further prospective studies and clinical trials are essential to provide a strong evidence-based recommendation that will help prolong the lifespan of patients.

## Introduction and background

The pancreas is an integral organ of our body performing two main functions: exocrine and endocrine functions [1]. Pancreatic cancer comprises 3% of all cancers and 7% of cancer-related deaths in the United States [1]. By the year 2030, pancreatic cancers are anticipated to be the second most common cause of cancer-causing deaths [2]. A primary malignant neoplasm can arise either in the exocrine pancreas or in the endocrine cells. Ductal adenocarcinoma is the most common type comprising 75-92% of all pancreatic tumors, where patients usually present at advanced stages and have a low survival rate [3]. For all stages combined, even with available treatment options like resection and chemotherapy, due to the late presentation with advanced cancer stage, the five-year relative survival is only 10% [1].

Type II diabetes mellitus (DM) is linked with pancreatic cancer through a complex association [4]. In most patients with pancreatic cancer, diabetes is diagnosed either concomitantly with cancer or within two years before the cancer diagnosis [5]. The association is not noted between long-standing DM and pancreatic cancer, but rather the association is found to be chiefly due to diabetes of recent onset, supposedly caused by the tumor itself [5]. An important point that is to be considered in the elderly presenting with DM is that new-onset diabetes may be a preliminary marker of pancreatic cancer [6]. Pancreatic cancer can result in DM either by damaging islet cells or by triggering insulin resistance. Such peripheral insulin resistance mostly occurs early in the disease course, which may illustrate why diabetes can manifest before the symptoms of the pancreatic tumor [5].

**Figure 1:**
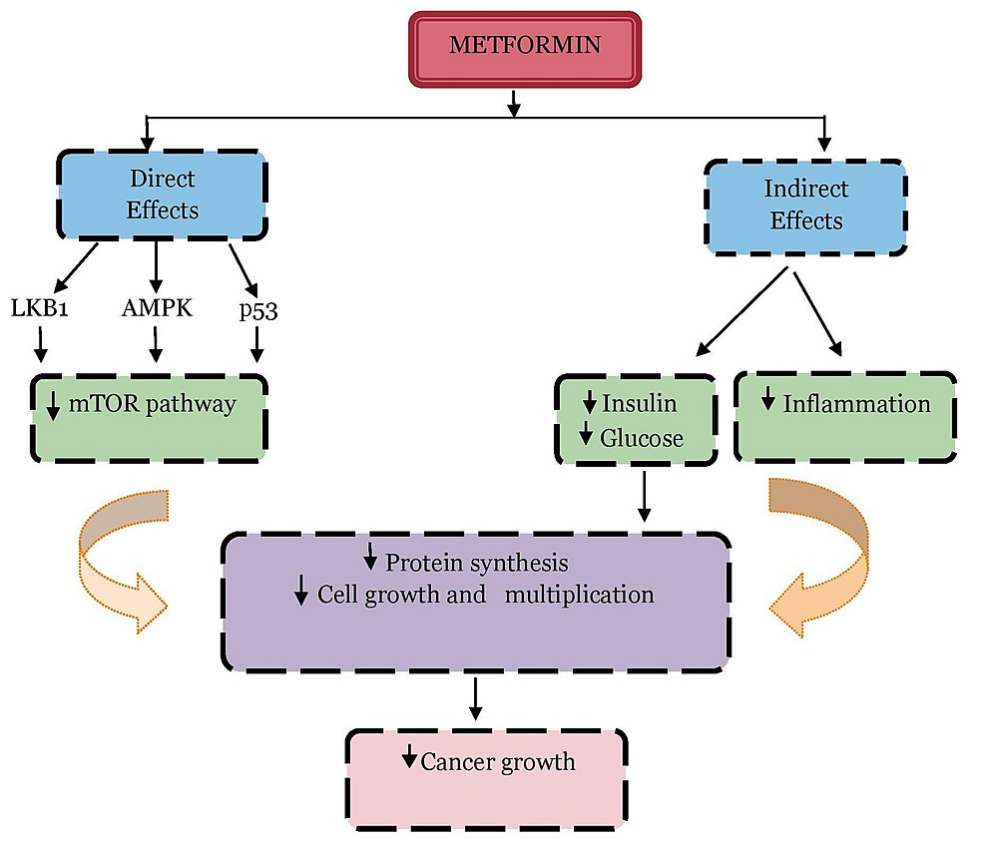
Mechanism of action of metformin in cancer growth AMPK: 5' adenosine monophosphate-activated protein kinase, LKB1: liver kinase B1, mTOR: mammalian target of rapamycin, p53: tumor protein p53.

Metformin, a biguanide group of oral hypoglycemic drugs, is one of the most widely used drugs for DM [7]. However, metformin is now known for its anti-neoplastic properties [8]. Metformin works by decreasing insulin resistance, and it also has direct growth-inhibitory action on various cancer cells [9]. Many studies have suggested that it acts directly by liver kinase B1 (LKB1)-dependent and 5′ adenosine monophosphate (AMP)-activated protein kinase (AMPK)-dependent suppression of the mammalian target of rapamycin (mTOR) pathway which inhibits protein formation and hence tumor cell multiplication and indirectly by decreasing insulin-mediated tumor growth and through its anti-inflammatory action [10-12]. The mechanism is illustrated in Figure 1. A large cohort study done in 2009 in patients in the United Kingdom diagnosed with DM showed that cancer was diagnosed among 7.3% of metformin users compared with 11.6% of non-users, thus showing reduced cancer risk with its use [13]. 

The objective of this systematic review is to further explore the efficacy of metformin in increasing the overall survival of patients with pancreatic cancer and concurrent DM. We included patients with pancreatic cancer and concurrent DM regardless of their age, gender, or country. The primary outcome was overall survival (OS). Information regarding disease-free survival (DFS), progression-free survival (PFS), and disease-specific survival (DSS) was also considered in this review. 

## Review

Methods

Study Protocol

We implemented Preferred Reporting Items for Systematic Review and Meta-Analyses (PRISMA) 2020 Guidelines [14], and accordingly, a systematic review was accomplished.

*Sources of Data Collection* 

We reviewed scientific literature from three databases, PubMed, Science Direct, and Scopus, using keywords and medical subject heading (MeSH) keywords from April 1 to April 3, 2021. MeSH strategy was used in PubMed, while keywords were mainly used in the other databases.

Search Strategy

The keywords used in our search are as follows: Pancreatic cancer, Diabetes Mellitus, and Metformin. In PubMed, the final search strategy with keywords and MeSH used was as follows: Pancreatic cancer OR Adenocarcinoma of pancreas OR neoplasms of pancreas ( "Pancreatic Neoplasms/drug effects"[Mesh] OR "Pancreatic Neoplasms/drug therapy"[Mesh] OR "Pancreatic Neoplasms/mortality"[Mesh] OR "Pancreatic Neoplasms/prevention and control"[Mesh] OR "Pancreatic Neoplasms/therapy"[Mesh] ) AND Diabetes Mellitus OR DM OR Type 2 DM ( "Diabetes Mellitus/drug therapy"[Mesh] OR "Diabetes Mellitus/mortality"[Mesh] OR "Diabetes Mellitus/prevention and control"[Mesh] OR "Diabetes Mellitus/therapy"[Mesh] OR "Diabetes Mellitus/complications''[Mesh] ) AND Metformin OR Biguanides OR Metformin hydrochloride( "Metformin/administration and dosage"[Mesh] OR "Metformin/adverse effects"[Mesh] OR "Metformin/therapeutic use"[Mesh] ). Databases with the used keywords are mentioned in Table 1.

**Table 1:** Databases and search strategy

Databases	Keywords	Search Results
PubMed	Final strategy as above	1,415
Science Direct	Pancreatic cancer and Diabetes Mellitus and Metformin	213
Scopus	Pancreatic cancer and Diabetes Mellitus and Metformin	345

Inclusion and Exclusion Criteria

Inclusion criteria were as follows: 1) Studies done in the last five years: from 2016 to April 2021. 2) Studies including clinical trials and randomized controlled trials (RCTs). 3) Studies with free full-text access. 4) Studies published in the English language only. 5) Studies performed only in humans. One article from the references was found relevant and was included. We excluded any studies done before 2016, animal studies, duplicate studies, grey literature, studies pertaining to all cancers but not only pancreatic cancer, and studies with unavailable full texts.

Data Extraction

We extracted data from included studies using standard data extraction form, and the data extracted was recorded under the following headings: authors, year of publication, country of study, sample size, stage of cancer, criteria for metformin user, other therapy, and outcome data including OS, DFS, PFS, and DSS in the intervention group. 

Risk and Quality Assessment

Two reviewers (MG and NV) individually obtained and evaluated the quality of the included articles. Newcastle Ottawa Scale was applied for observational studies and Revised Cochrane’s Risk of Bias Tool for RCTs. Only those screened articles that satisfied >70% of the checklist quality parameters were included in our study.

Results

**Figure 2:**
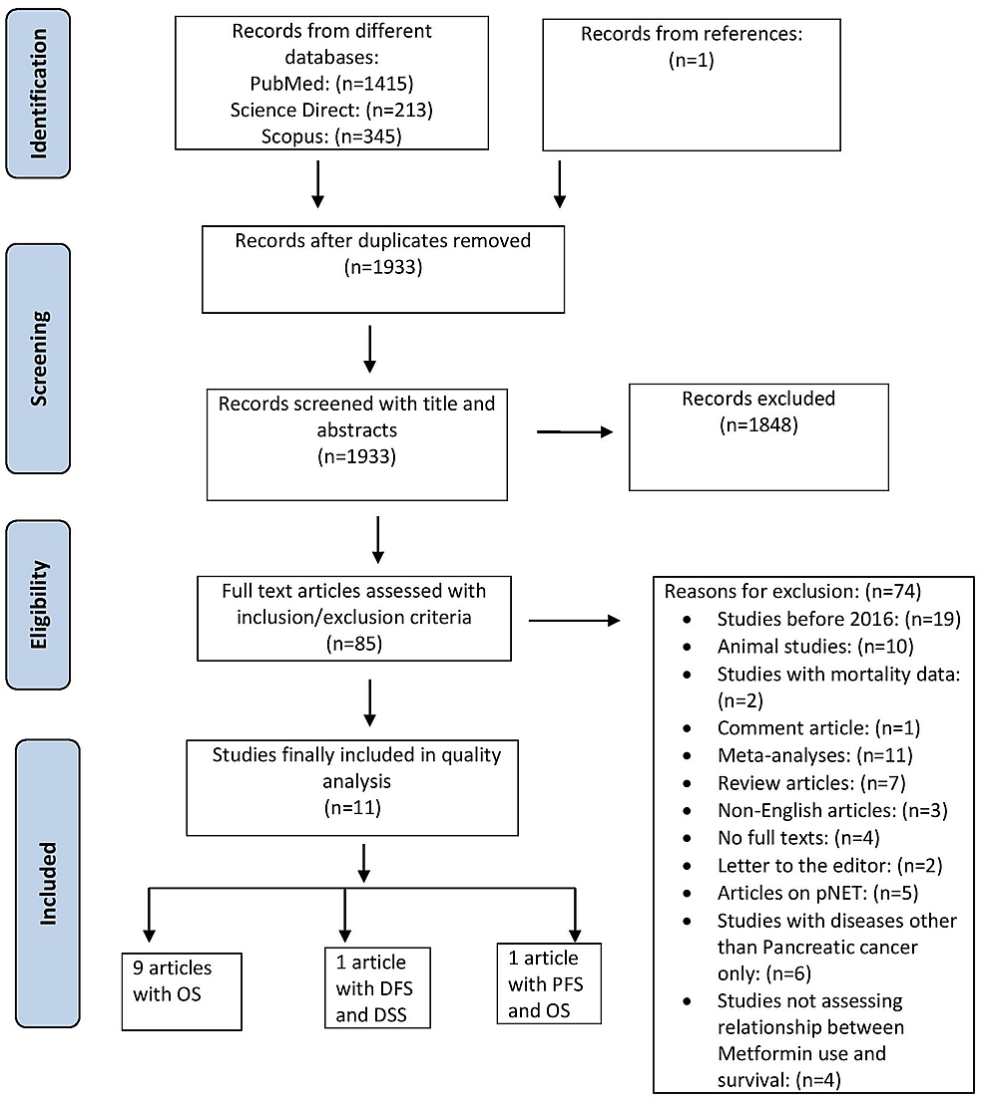
PRISMA flow diagram DFS: Disease-free survival, DSS: disease-specific survival, OS: overall survival, PFS: progression-free survival, PRISMA: Preferred Reporting Items for Systematic Reviews and Meta-Analyses.

A total of 1,973 articles were identified after applying our search strategies: 1,415 from PubMed, 213 from Science Direct, and 345 from Scopus. A total of 1,933 articles remained after excluding the duplicates. The articles were then screened based on the title and abstract. The articles were also filtered according to the full-text content and the inclusion/exclusion criteria. Finally, a quality check was performed based on the study characteristics, after which 11 articles remained at the end. Among these final articles, nine studies reported data for OS, one for DFS/DSS, and one for PFS/OS. Lastly, a PRISMA flow diagram was created, as shown in Figure 2.

Study Characteristics

Our review included patients from 10 cohort studies and one RCT. Table 2 shows the characteristics of the included studies.

**Table 2:** Study characteristics APC: Advanced pancreatic cancer, DFS: disease-free survival, DM: diabetes mellitus, DPP4: dipeptidyl peptidases-4, DSS: disease-specific survival, N: no, NA: not available, NR: not reported, OS: overall survival, PC: pancreatic cancer, PDAC: pancreatic adenocarcinoma, PFS: progression-free survival, RCT: randomized controlled trial, TZD: thiazolidinediones, Y: yes.

Author	Year	Country	Study Design	Sample Size	Stage of Cancer	Metformin User Criteria	Other Therapy	Survival Analysis	Conclusions	Survival Benefit
Tamburrino et al. [15]	2020	Italy	Retrospective cohort	430	Resectable PC	Started drug at least six months before diagnosis	NR	DFS and DSS	Metformin was associated with better DFS without statistical significance, and no DSS improvement was observed.	N
Terasaki et al. [16]	2020	Japan	Retrospective cohort	373	Resectable PDAC	The drug was taken >90 days before cancer diagnosis	Insulin, sulfonylurea, TZD, alpha-glucosidase inhibitors	OS	OS was observed to be better in the metformin treatment group compared to other medications, with a five-year survival rate significantly better in the metformin group.	Y
Dulskas et al. [17]	2020	Lithuania	Retrospective cohort	454	PC	Prescription requests at any time	Insulin, sulfonylurea, TZD, others	OS	Improved survival was observed in the patient groups using metformin and in combinations of metformin with other anti-hyperglycemics.	Y
Toriola et al. [18]	2019	United States	Retrospective cohort	3,811	All stages of PDAC	Prescription requests at any time	Insulin, sulfonylurea, TZD, alpha-glucosidase inhibitors, DPP4 inhibitors, meglitinide	OS	No associations between metformin usage and survival in patients with PDAC were found, but there appeared to be a survival benefit in non-Hispanic White patients who were metformin naive at the time of diagnosis.	N
Frouws et al. [19]	2017	Netherlands	Retrospective cohort	907	All stages of PC	Use of drug at any time for at least 30 days	Sulfonylurea	OS	No interconnection was observed between OS, pancreatic cancer, and metformin use.	N
E et al. [20]	2017	United States	Cohort	5,621	PDAC	Usage of drug at least three months before diagnosis	Insulin	OS	Significant benefits of statins but not metformin on survival among the elderly PDAC patients were seen.	N
Lee et al. [21]	2016	Korea	Retrospective cohort	237	All stages of PC	Using metformin at the time of diagnosis	Insulin, sulfonylurea, TZD, DPP4 inhibitors	OS	Metformin usage was associated with survival benefits in patients with pancreatic cancer with pre-existing type II DM, particularly among those with an advanced stage of cancer.	Y
Choi et al. [22]	2016	Korea	Retrospective cohort	349	Advanced PC	Usage of the drug at DM diagnosis	Insulin, sulfonylurea	OS	Metformin treatment correlates with longer OS in APC patients undergoing palliative chemotherapy.	Y
Cerullo et al. [23]	2016	United States	Retrospective cohort	3,393	Resectable PC	Prescription proof	NA	OS	Treatment with metformin was linked with an increased OS in patients undergoing a surgical procedure for pancreatic cancer.	Y
Chaiteerakij et al. [24]	2016	United States	Retrospective cohort	980	All stages of PDAC	Usage of the drug at DM diagnosis	NA	OS	The findings did not indicate the benefit of metformin after diagnosis of PDAC patients.	N
Reni et al. [25]	2016	Italy	RCT	60	Metastatic PC	Drug intake after cancer diagnosis	Chemotherapy	PFS, OS	Results showed that supplementation of metformin at the dose prescribed for DM did not raise the survival outcome in metastatic pancreatic cancer patients receiving standard systemic therapy.	N

Discussion

While reviewing all 11 articles, we tried to determine the usefulness of metformin treatment in diabetic pancreatic cancer patients.

In pancreatic cancer, associated type II DM is prevalent and the association is complex [4]. Pancreatic cancer-associated diabetes is most probably due to the combination of beta-cell dysfunction and insulin resistance [26]. Since most cases of pancreatic cancer are advanced and incurable [27], many kinds of researches have been done regarding drugs that can improve survival in diabetic pancreatic cancer patients [28]. Although there is a positive effect between metformin use in pancreatic cancer patients with diabetes and overall survival, the opposite has also been reported. Many articles reveal positive effects [29-32], whereas some reveal no positive effects [33,34]. 

Tamburrino et al. retrospectively analyzed a population of 430 patients with resectable pancreatic cancer in Italy and found that metformin users had a three-year DFS of 52% versus 34% in non-users, concluding that metformin is not associated with better survival (p=0.083) [15]. However, another retrospective study by Terasaki et al. on resectable pancreatic cancer revealed that the five-year survival rate was superior in the metformin group as opposed to other medications groups (66.7% and 24.4%, respectively, with p=0.034) [16].

Meanwhile, Dulskas et al. considered 454 patients with pancreatic cancer and found that better survival existed in the metformin users’ group (p=0.003), and in the multivariate analysis, the death rate of metformin users was found to be lower than that of other anti-hyperglycemic users, but it was insignificant (HR: 0.74, 95% CI: 0.54-1.02, p=0.07) [17]. Chaiteerakij and colleagues evaluated 980 diabetic pancreatic cancer patients to detect that metformin did not cause any survival benefit in various sensitivity analyses (HR: 0.93, 95% CI: 0.81-1.07, p=0.28) [24].

Lee et al. conducted a cohort study in South Korea, and in both univariate and multivariate testing, metformin exposure was coupled with a greater survival interval (13.7 months for metformin versus 8.9 months for non-metformin users) [21]. Also, positive survival improvement was seen in the advanced pancreatic cancer group (HR: 0.61 and p=0.001) [21]. A separate retrospective study by Choi et al. in Korea found that metformin treatment caused better OS as to other oral hypoglycemic drugs within diabetic patients (HR: 0.69, 95% CI: 0.49-0.97, p=0.036) and also in the advanced stage of pancreatic cancer (HR: 0.697, 95% CI: 0.491-1.99, p=0.04) [22].

Additionally, a large cohort study by Toriola et al. with 3,811 patients with entire stages of pancreatic cancer concluded that metformin use did not improve the overall survival [18]. But, in patients with no metformin use at the time of diagnosis when stratified by race, enhanced survival was observed in the non-Hispanic White population (HR: 0.78, 95% CI: 0.61-0.99, p=0.04) but not in the African American population (HR: 1.20, 95% CI: 0.75-1.93, p=0.45) [18]. E et al. revealed that metformin use did not correlate with the overall survival improvement in older pancreatic ductal adenocarcinoma patients in neither pre-diagnosis users (HR: 1.02, 95% CI: 0.97-1.06, p=0.52) nor post-diagnosis users (HR: 0.99, 95% CI: 0.87-1.13, p=0.88) [20]. Similarly, an observational cohort study revealed that after balancing confounders, no significant survival benefit was seen in metformin users compared to non-users (RR: 0.86, 95% CI: 0.66-1.12, p= 0.23) [19].

In 2016, Cerullo et al. surveyed 3,393 patients and found that metformin usage improved the OS after 18 months of cancer resection irrespective of other received adjuvant therapies (OS at 18 months of surgery with metformin: 59.6% versus 53.4% without metformin, p=0.012) [23]. Reni et al. performed a randomized phase II trial with 60 patients and revealed no advantage in OS and PFS after adding metformin to a chemotherapy regimen of cisplatin, epirubicin, capecitabine, and gemcitabine (PEXG) in metastatic pancreatic cancer [25]. This result was consistent with another placebo trial performed by Kordes et al. that showed no outcome improvement with metformin usage when added to standard therapy of gemcitabine and erlotinib in advanced pancreatic cancer [35].

Limitations

Limitations of this systematic review include reviewing many observational studies with just one randomized clinical trial and no case-control studies. So, stronger evidence-based studies are missing. Also, owing to the observational method of included studies, the consequences of confounding could not be dismissed. As the populations differed in staging, concurrent treatment, the relative time of metformin exposure with no appropriate dosage information, and follow-up period, the results should be interpreted carefully.

## Conclusions

We studied different articles that evaluate the association between metformin when used in diabetic patients with pancreatic neoplasm and overall survival outcomes. Eventually, we found that metformin has been shown to have augmented survival in some studies, whereas no improvement was observed in other studies. The benefits of metformin versus no metformin treatment are to be considered thoughtfully in diabetic pancreatic cancer patients. To establish the positive effects of metformin in diabetic patients with pancreatic cancer, we need to conduct more clinical trials with larger study populations to further investigate the benefits that can then help future health care providers improve patient outcomes.
